# Case report: Rehabilitation course in thrombocytopenia, anasarca, fever, reticulin fibrosis/renal failure, and organomegaly syndrome complicated by cerebral infarction in the left parabolic coronary region

**DOI:** 10.3389/fneur.2023.1153941

**Published:** 2023-07-13

**Authors:** Takamasa Hashizaki, Yukihide Nishimura, Tokio Kinoshita, Kohei Minami, Makoto Kawanishi, Yasunori Umemoto, Fumihiro Tajima

**Affiliations:** ^1^Department of Rehabilitation Medicine, Wakayama Medical University, Wakayama, Japan; ^2^Division of Rehabilitation, Wakayama Medical University Hospital, Wakayama, Japan; ^3^Department of Rehabilitation Medicine, Iwate Medical University, Shiwa-gun, Japan

**Keywords:** thrombocytopenia anasarca fever reticulin fibrosis/renal failure organomegaly syndrome, stroke, rehabilitation, Borg scale, case report

## Abstract

Although thrombocytopenia, anasarca, fever, reticulin fibrosis/renal failure, and organomegaly (TAFRO) syndrome was first reported in 2010, its pathogenesis and prognosis are still unknown. Moreover, reports on rehabilitation in patients with TAFRO are limited. In severe cases, dyspnea and muscle weakness could impede improvements in activities of daily living (ADL). However, reports on exercise intensity showed no worsening of TAFRO within the load of 11–13 on the Borg scale. Herein, we describe the rehabilitation and progress in a 61-year-old woman with TAFRO syndrome complicated by cerebral infarction from early onset to discharge. After cerebral infarction onset in the perforating artery, she was admitted to the intensive care unit due to decreased blood pressure and underwent continuous hemodiafiltration. Two weeks following transfer to a general ward, the patient started gait training using a brace due to low blood pressure, respiration, and tachycardia. After initiating gait training, increasing the amount of training was difficult due to a high Borg scale of 15–19, elevated respiratory rate, and worsening tachycardia. Furthermore, there was little improvement in muscle strength on the healthy side after continuous training, owing to long-term steroid administration. On day 100 after transfer, the patient was discharged home with a T-cane gait at a monitored level. The patient had severe hemiplegia due to complications with severe TAFRO syndrome delaying early bed release and gait training; tachycardia; and respiratory distress. Additionally, delayed recovery from muscle weakness on the non-paralyzed side made it difficult for the patient to walk and perform ADLs. Despite these issues, low-frequency rehabilitation was useful. However, low-frequency rehabilitation with gait training, using a Borg scale 15–19 orthosis, did not adversely affect the course of TAFRO syndrome.

## 1. Introduction

Thrombocytopenia, anasarca, fever, reticulin fibrosis/renal failure, and organomegaly (TAFRO) syndrome is a systemic inflammatory disease with a currently unknown cause (1, 2). Treatment of TAFRO includes steroids and immunosuppressive therapy (3). Additionally, its complications include cerebral infarction due to an abnormal blood coagulation system, interstitial lung lesions, and dilated cardiomyopathy (4, 5). According to the 2019 diagnostic criteria, TAFRO is diagnosed based on: (i) fluid retention (pleural effusion, ascites, and generalized edema); (ii) thrombocytopenia (100,000/μl); (iii) fever of unknown origin >37.5°C or serum C-reactive protein (CRP) concentration of 2 mg/dl. Moreover, the minor criteria for establishing this diagnosis comprise Castleman's disease-like findings on lymph node biopsy; bone marrow fibrosis; mild organ enlargement (hepatosplenomegaly or lymphadenopathy); and progressive renal involvement.

The disease severity is rated on a 3-point scale regarding each of the four categories: asthenia including pleural effusion and ascites; thrombocytopenia; fever and inflammation; and renal failure. Depending on the total score, severity is classified as: 0–4 points, mild (grade 1); 5–6 points, moderate (grade 2); 7–8 points, somewhat severe (grade 3); 9–10 points, severe (grade 4); and 11–12 points, very severe (grade 5) (6).

Although reports describing rehabilitation in patients with TAFRO syndrome are limited, dyspnea caused by thoracoabdominal effusion and muscle weakness is thought to impede rehabilitation progress. Improvement in activities of daily living (ADL) is also challenging to achieve in severe cases (7, 8). Furthermore, aerobic and strength exercises within the Borg scale rate of perceived exertion (RPE) of 11–13 do not appear to cause adverse events or exacerbate pathological conditions (8). The guidelines for cerebrovascular disorder treatment recommend early release from bed and early gait training with orthotics for rehabilitation (9). However, to date, there are no reports on early rehabilitation in patients with TAFRO syndrome complicated by cerebral infarction. In addition, whether gait training with orthotics exacerbates the condition remains unclear.

In this study, we attempted gait training using an orthotic device in a patient with severe right hemiplegia due to perforator artery infarction with severe TAFRO syndrome to wean the patient from continuous hemodiafiltration (CHDF) and reacquire gait. We assessed the contents of training and difficulties in practicing in each stage of the disease; progress of improvement in motor function and ADL, and whether early rehabilitation had any adverse effects on TAFRO syndrome based on blood test results.

## 2. Case report

**Figure 1:**
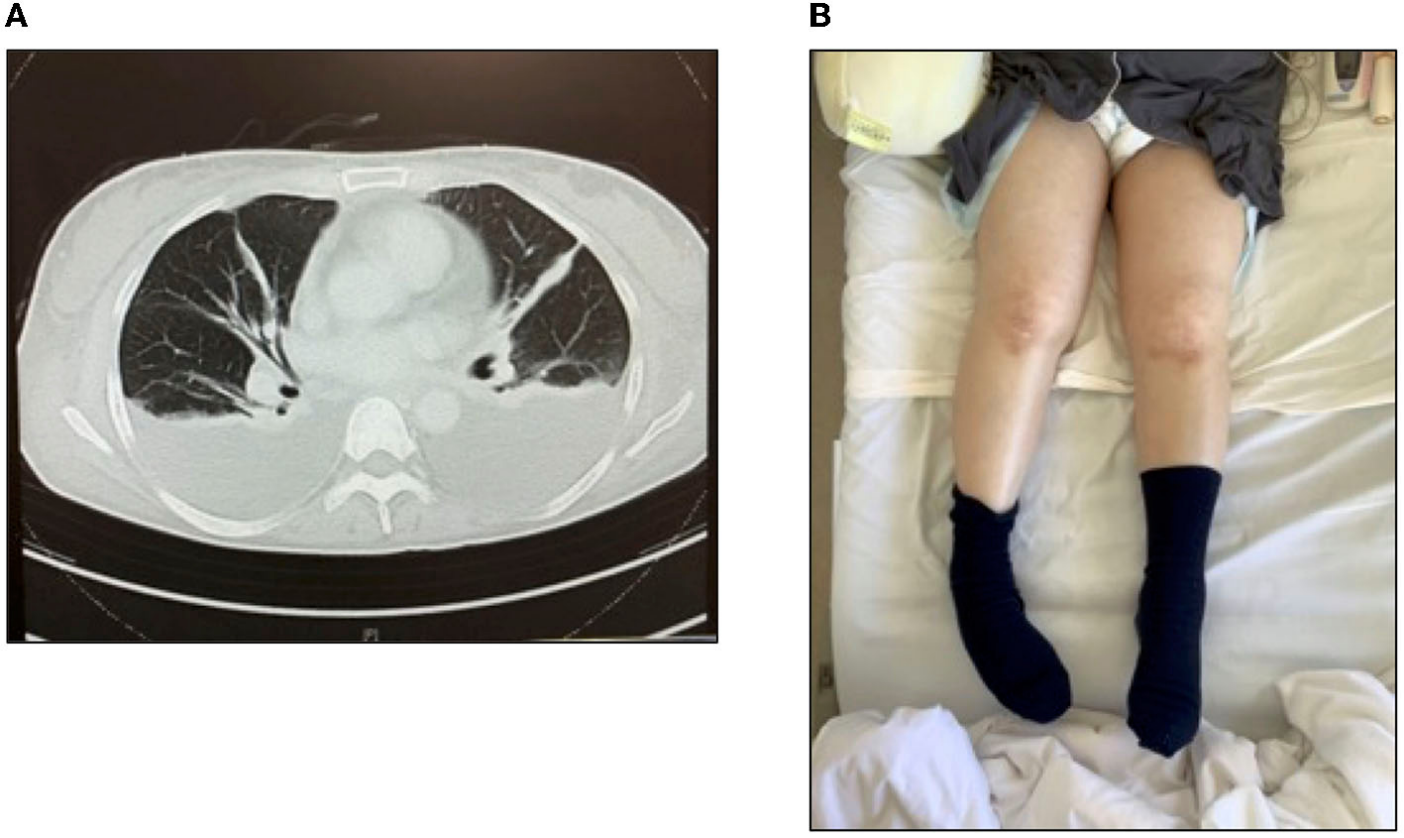
Thoracic ascites and generalized edema and the diagnostic criteria for TAFRO syndrome. **(A)** Computed tomography image of the chest and **(B)** generalized edema.

The patient was a 61-year-old woman with no remarkable medical history and independent ADL before her illness. She experienced coronavirus disease 2019 and was admitted to a community hospital, after which she was discharged home. However, the patient was admitted again on suspicion of cholecystitis and transferred to our university hospital due to a persistent inflammatory reaction and acute renal failure. The patient was treated with steroids (1,000 mg/day) and hemodialysis (HD). Moreover, physical therapy was initiated the following day after admission. Only indoor rehabilitation was provided to prevent post-coronary infection. On day seven after admission, we observed fluid retention (pleural and ascites effusions, generalized edema; Figures 1A, B), platelet loss (PLT), fever, high CRP levels, and bone marrow fibrosis: the essential diagnostic criteria for TAFRO syndrome. Creatinine (Cre) levels, an indicator of renal dysfunction, were also elevated. The patient was diagnosed with TAFRO syndrome secondary to bone marrow fibrosis, mild organ enlargement, and progressive renal impairment. On day 12 after hospitalization, another pulse of steroids was administered (1,000 mg/day). However, the symptoms did not resolve. On day 13, chest pain appeared (no enzyme excursion or abnormal electrocardiogram), and pleural effusion increased.

**Figure 2:**
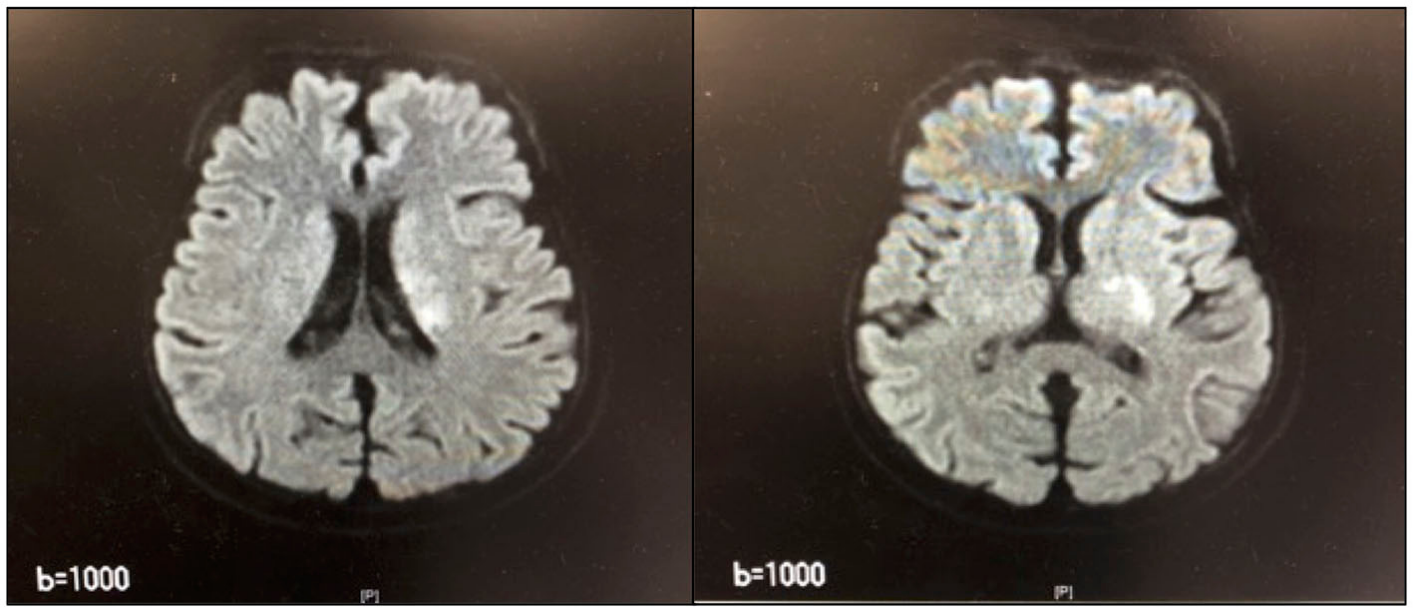
Magnetic Resonance Imaging of the head. High-signal area from the left parabasal corona to the inner retrosternal leg.

After 14 days, she developed branch atheromatous disease (BAD; Figure 2) in the perforating artery of the left radio coronary artery. The patient was treated conservatively but was admitted to the intensive care unit (ICU) on day 15 for CHDF due to low blood pressure (BP). In the ICU, the patient received CHDF, range-of-motion training of the limbs while in bed due to the low BP, as well as muscle-strengthening exercises of the limbs of the healthy side. Additionally, on day 19, the patient was weaned from CHDF, transferred to HD, and discharged from the ICU.

On discharge from the ICU, the patient's TAFRO syndrome severity classification was grade 4 (severe) (6), with resting heart rate (HR; 108), BP (111/82), and oxygen saturation (SpO_2_; 96%). Physical examination revealed abdominal distention due to pleural effusion and marked edema in the trunk, both lower extremities, and right upper extremity. Although the patient was conscious and had good communication without obvious higher brain dysfunction, she had difficulty articulating. Muscle strength on the manual muscle testing (MMT) (10) was 0 in the right upper and lower limbs (no muscle contraction at all) and 4 in the left upper and lower limbs (full range of motion overcoming gravity even with some resistance), with severe right hemiplegia and muscle weakness on the non-paralytic side. The patient required heavy-to-full assistance when getting up, sitting on the edge, or standing up. Her HR also increased over 130 while sitting and standing. Although there was no evidence of tachycardia or decreased SpO_2_, she could only maintain sitting in a wheelchair for ~5 min with or without assistance due to respiratory distress and tachypnea (upper 20 to lower 30 per min). She was administered 45 mg of methylprednisolone as a steroid. The goal of physiotherapy was to improve the patient's endurance in a wheelchair position and start gait training rehabilitation with an orthosis promptly. Rehabilitation consisted of sitting for 5 min, standing for 1–2 min, and 5–10 min in the wheelchair transfer position. The patient's tachycardia and dyspnea gradually decreased, and she could sit in a wheelchair for ~20 min.

**Figure 3:**
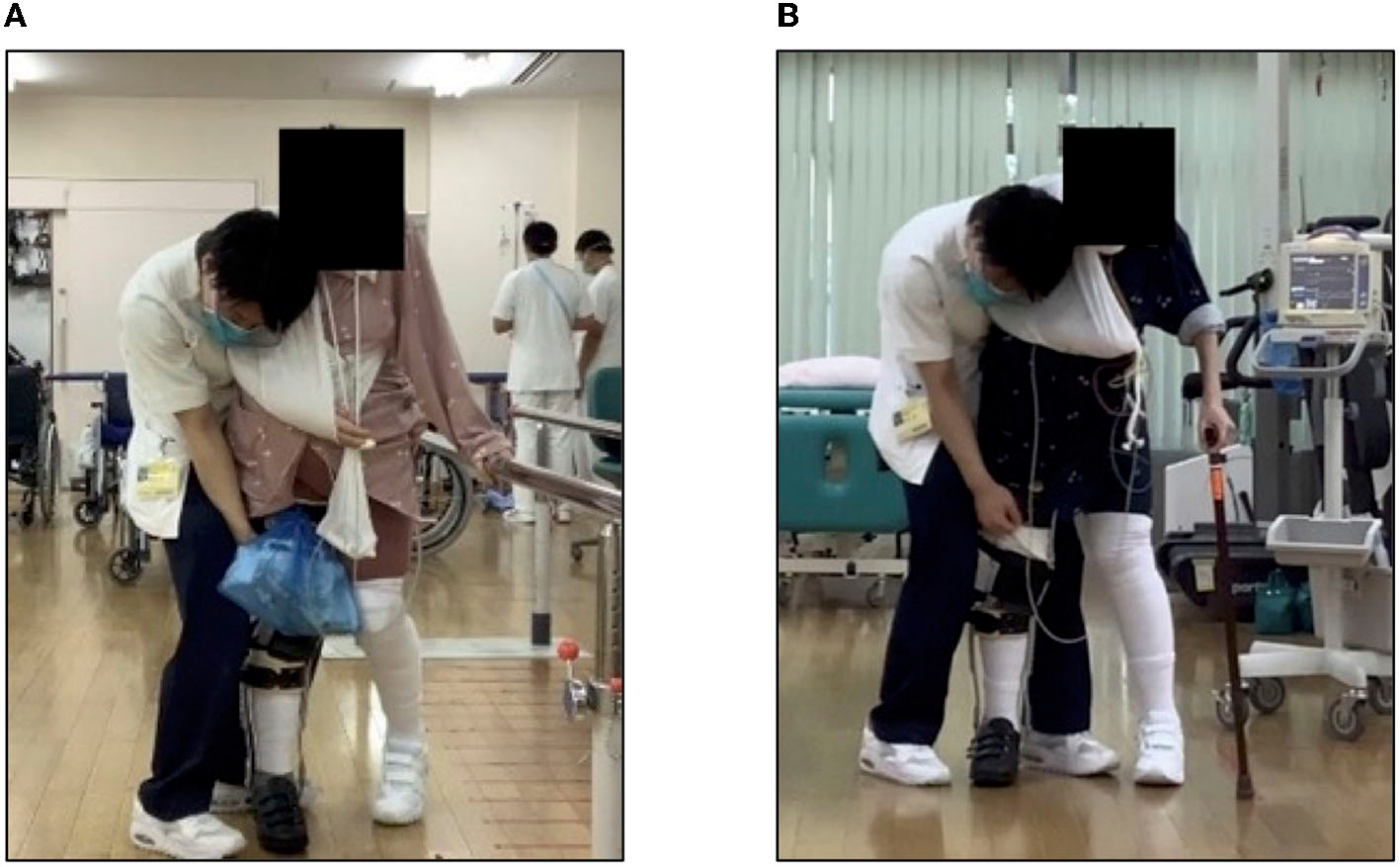
Gait training using long leg brace. **(A)** Walking with parallel bars and **(B)** walking with a T-joint cane.

On day 27 after admission, the patient was transferred for gait training using a long leg brace (LLB). She started walking on the parallel bars. However, after ~5 min, her HR increased from 100 to 150 beats/min, her resting respiratory rate increased from 15 to 30 beats/min, experiencing severe respiratory distress. The patient's subjective exercise intensity was Borg RPE 15 (hard)−19 (very hard; Figure 3A). The patient could perform only four sets of 10 m walk over 60 min of training. The patient was transferred to a community hospital on day 56 due to tachycardia and respiratory distress. Gait training could not be gradually increased. At the time of transfer, her gait training consisted of three sets of 10 laps on parallel bars (three sets of 60 m) and three sets of 15 m continuous T-cane walking (Figure 3B). The steroid dose was also reduced to 30 mg of prednisolone (PSL).

On day 71, she was transferred back to our hospital due to hemorrhage from a rectal ulcer and underwent surgical suturing. After 75 days, the Hematology Department requested rehabilitation, and walking training was resumed on day 76. The patient's muscle strength in the right lower limb did not recover to MMT 0. Therefore, she was provided with walking assistance using LLB. On day 77, her respiratory rate was over 30 after ~100 m of continuous T-cane walking. On day 86, there was no significant change in motor paralysis of the right limbs. The patient was in a state of light transfer and moderate walking assistance. Assuming the patient would live at home, LLB was cut down and gait training with a short-leg orthosis began. During a 60 m T-cane assisted walk, the patient's HR was over 120, respiratory rate was ~20 breaths/min, and Borg RPE was 12–13. On day 100, the patient was discharged home.

**Figure 4:**
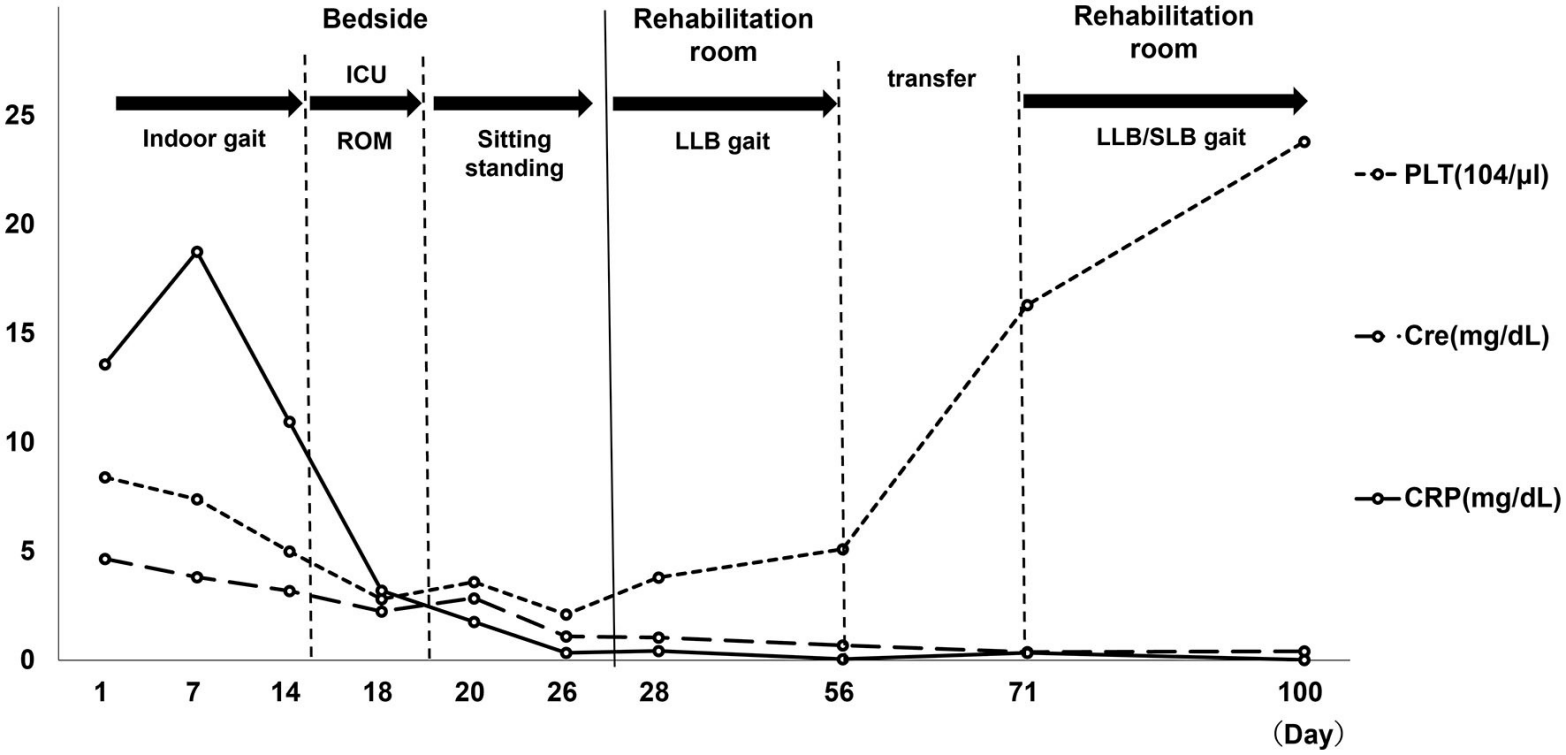
Blood collection item. PLT, platelet; Cre, creatinine; CRP, C-reactive protein; ROM, range of motion; LLB, long leg brace; ICU, intensive care unit. Day 1: hospitalization, Day 7: TAFRO diagnosis, Day 14: Cerebral infarction Onset. Day 19: Start of bed release, Day 27: Start of LLB gait, Day 56: hospital transfer. Day 100: discharge from hospital.

On discharge, PLT (23.8 × 104 μl), CRP (0.02 mg/dl), and renal impairment improved, without pleural or ascites effusion. The patient was classified as grade 1 on the TAFRO syndrome severity scale (6). Additionally, resting HR was in the 70s, MMT was 1 in the right limbs (slight muscle contraction was observed) and 4 in the left limbs. However, muscle weakness persisted on the healthy side. The patient's basic activities were independent getting up and transferring to a wheelchair using a motorized bed, independent T-cane walking with minimal assistance, and continuous walking distance of ~100 m. The subjective exercise intensity was Borg RPE 13 (slightly severe), HR was 110–120 during exertion, and respiratory rate was 15–20 breaths/min: improvement was observed. The functional independence measure (FIM) was 47/126 at intervention, 58/126 at transfer, and 82/126 at discharge. Moreover, the PSL dose at discharge was 12 mg. A summary of the blood test results throughout the patient's admission time is shown in Figure 4.

When first diagnosed with TAFRO syndrome, CRP and Cre levels were elevated, and her PLT levels had decreased. Elevated CRP levels persisted after the onset of cerebral infarction. When released from the hospital ward, blood samples showed worsening of PLT, CRP, and Cre levels. Moreover, when starting gait training using LLB, her Borg RPE was 15–19 and HR over 150. She continued rehabilitation with HR over 150 and tachypnea (30 breaths/min). However, there was no deterioration in blood test results by the day 56 after hospital transfer. Thereafter, no further deterioration in blood test results was noted until discharge home on day 100.

Written informed consent was obtained from the patient's family to publish this case report. This study conforms to all case report guidelines.

## 3. Discussion

This study reported the rehabilitation progress in a patient with severe TAFRO syndrome and right hemiplegia due to BAD. The patient had difficulty in early release from bed and participating in rehabilitation due to tachycardia, low BP, and respiratory distress. In addition, the patient had prolonged muscle weakness on the healthy side, making it difficult to resume ADL. Furthermore, the rehabilitation load after the patient could leave bed was considered high-intensity based on the HR and Borg RPE results. However, providing a high-intensity exercise load at a low frequency did not seem to adversely affect the patient's general condition or blood test results. To our best knowledge, this is the first case report to describe, in details, the content and course of rehabilitation treatment in a patient with TAFRO syndrome complicated by cerebral infarction.

In a previous study on patients admitted to the ICU, those out of bed within 24 h after admission had a significantly shorter time to gain standing and walking abilities than did those in the standard physical therapy group. Furthermore, their ADL improvement at discharge was also significantly better (11). Out of bed within 24 h has been reported to increase ADL improvement in patients with cerebrovascular disease at the time of transfer to an acute care hospital and 6 months later, with most improvements in exercise abilities (12, 13). Therefore, early release from the ICU is recommended for patients with a cerebrovascular accident, even under ICU management However, the rehabilitation expert consensus recommends withholding active exercise in the case of unstable cardiac rhythms and supplemental circulation, such as an intra-aortic balloon pump, or if BP was too low, even with high doses of inotropic and hypertrophic drugs (14).

TAFRO syndrome is associated with cardiomyopathy. Echocardiography has shown decreased wall motion of the left ventricle, which may require inotropic therapy or left ventricular assist (4). Cytokine storms caused by TAFRO syndrome have been also reported to cause systemic edema and intravascular dehydration associated with cardiac complications (15). In the present case, after complications of BAD, echocardiography showed no decrease in wall motion (ejection fraction, 59 %). However, BP was low due to systemic edema and intravascular dehydration. Additionally, the patient's circulatory and general condition was severe enough for the ICU, including CHDF. Moreover, the Japanese stroke treatment guidelines recommend gait training with orthotics at an early stage (9). Even after discharge from the ICU, the patient had tachycardia and dyspnea, considered TAFRO syndrome symptoms, and took ~2 weeks before being able to sit in a wheelchair and begin gait training in a rehabilitation room. A previous study reported that in terms of TAFRO severity, patients with thoracoabdominal effusion, respiratory distress, tachypnea, and abdominal distention appeared even in a gaggle-up sitting position, which inhibited the progress of rehabilitation (7). This case is similar to previous research, and it appears that patients with severe TAFRO syndrome might have a significant delay in appropriate training, such as bed release and gait training.

A large amount of rehabilitation is recommended for improving ADL according to the Japanese guidelines for people with cerebrovascular disorders (9). Previous studies have reported a correlation between total daily rehabilitation time and functional improvement, with longer time contributing to the improvement in total FIM scores (16). However, in the present case, tachycardia and respiratory distress appeared even after the start of gait training. Therefore, it was difficult to secure the time and distance for gait training. According to the criteria for discontinuation of rehabilitation in Japan, a pulse rate exceeding 140 beats/min is considered a threshold for termination in the middle of a rehabilitation program (17).

At our hospital, physiatrists may examine patients with cerebral infarction and continue gait training even with tachycardia exceeding 140 beats/min after risk management. However, in the present case, the patient had tachycardia of 150 beats/min during a 5 m assisted walk, and thoracoabdominal effusion due to TAFRO syndrome, with a respiratory rate exceeding 30 beats/min, resulting in respiratory distress and Borg RPE 15–19. In addition, it took 5–10 min for the respiratory rate and Borg RPE to return to a resting level, and the total walking distance was extremely short compared with patients with stroke in our hospital. Previous reviews reported that the 5-year survival rate of patients with TAFRO is severe (66.5%). Thus, timely diagnosis and rational treatment remain a major challenge for clinicians, and the prognosis and course of TAFRO syndrome are unclear (18). Even in those who respond well to drug treatment, symptoms can take at least one year to subside (19). Additionally, in severe TAFRO syndrome, it may be difficult to titrate the training dose over a long period of time due to the time required to recover from symptoms such as tachycardia and tachypnea. In patients with TAFRO syndrome and BAD, low BP, tachycardia, and respiratory distress may delay the start of early weaning and gait training. Moreover, difficulties may persist after initiation to maintain walking distance, shorten rehabilitation time, and improve ADLs.

The current patient still had muscle weakness in the healthy limbs at the time of discharge from the hospital, suggesting that disuse muscle weakness occurs due to the inability to secure a large amount of activity due to edema and respiratory distress. In addition, muscle weakness also may be due to steroid use (20, 21). Furthermore, prolonged muscle weakness on the healthy side may exacerbate delays in ADL recovery.

The effects of exercise on systemic inflammation and organ function in patients with TAFRO syndrome are unknown. In previous physical therapy, the most severely ill patients were trained to perform 10–20 consecutive Borg RPE 11–13 muscle strengthening exercises and walking endurance training. Adjusting the walking distance to 11–13 on the Borg RPE scale also resulted in improvement in bedridden state with a Barthel Index score of 0 to walking independently with an ankle-foot orthosis without adverse events or condition worsening (7). In the current case, the patient experienced cerebral infarction. Additionally, when orthotic-assisted gait training was performed, the exercise was Borg RPE 15–19. Compared with previous reports, high-intensity exercise did not exacerbate the symptoms of TAFRO syndrome if performed at a low frequency with long rest periods.

TAFRO syndrome is a relatively new disorder, first reported in 2010 (1). Therefore, the pathogenesis and prognosis remain unclear. Furthermore, since this study is a case report, it is difficult to generalize the course in this patient to other patients with TAFRO syndrome. However, this report may provide valuable information on rehabilitation and progress in patients with TAFRO syndrome complicated by cerebral infarction.

## 4. Conclusions

In this case of severe hemiplegia due to BAD combined with severe TAFRO syndrome, the patient was in a serious condition, with delayed early bed release and gait training initiation. In addition, delayed recovery from muscle weakness on the non-paralyzed side of the body made it difficult for the patient to walk and perform ADLs. However, the results suggest that low-frequency rehabilitation with Borg RPE 15–19 orthotic gait training did not have an adverse effect on the condition and could be useful for treating such patients.

## Data availability statement

The raw data supporting the conclusions of this article will be made available by the authors, without undue reservation.

## Ethics statement

Ethical review and approval was not required for the study on human participants in accordance with the local legislation and institutional requirements. The patients/participants provided their written informed consent to participate in this study. Written informed consent was obtained from the individual(s) for the publication of any potentially identifiable images or data included in this article.

## Author contributions

TH and YN conceptualized and designed the study, drafted the initial manuscript, and reviewed and revised the manuscript. TK, KM, MK, and YU designed the data collection instruments, collected data, carried out the initial analyses, and reviewed and revised the manuscript. FT designed the data collection instruments, coordinated and supervised the data collection, and critically reviewed the manuscript. All authors approved the final manuscript as submitted and agreed to be accountable for all aspects of the work.
